# Esketamine improves propofol-induced brain injury and cognitive impairment in rats

**DOI:** 10.1515/tnsci-2022-0251

**Published:** 2022-12-06

**Authors:** Guiping Xu, Yang Wang, Zhe Chen, Yuxuan Zhang, Xuexue Zhang, Guichao Zhang

**Affiliations:** Department of Anesthesiology, People’s Hospital of Xinjiang Uygur Autonomous Region, Xinjiang Clinical Research Center for Anesthesia Management, Urumqi 830001, China; Graduate School of Xinjiang Medical University, Urumqi 830000, China; Medical School, Shihezi University, Xinjiang, Shihezi, 832000, China

**Keywords:** brain injury, cognition impairment, esketamine, propofol, mBDNF/TrkB/PI3K signaling

## Abstract

As an intravenous anesthetic, propofol has been indicated to induce neurotoxicity in both animal and human brains. It is of great significance to better understand the potential mechanism of propofol-induced neurotoxicity to eliminate the side effects of propofol. Esketamine is a sedative that has been proven to have an antidepressant effect. However, its effect on propofol-induced neurotoxicity and the underlying mechanism remain unclear. Herein, we investigated the role of esketamine in propofol-induced brain injury. A rat model of propofol-induced brain injury was established with or without the treatment of esketamine. The results demonstrated that propofol-induced impairment in spatial learning and memory of rats and promoted oxidative stress, neuronal injury and apoptosis in rat hippocampal tissues. The effects caused by propofol were attenuated by esketamine. Esketamine activated the mature brain-derived neurotrophic factor/tropomyosin receptor kinase B/phosphatidylinositide 3-kinase (mBDNF/TrkB/PI3K) signaling pathway in propofol-administrated rats. Moreover, knocking down BDNF partially reversed esketamine-mediated activation of the mBDNF/TrkB/PI3K signaling pathway and inhibition of neuronal apoptosis in propofol-induced rats. Overall, esketamine mitigates propofol-induced cognitive dysfunction and brain injury in rats by activating mBDNF/TrkB/PI3K signaling.

## 1 Introduction

General anesthesia is an inevitable part of surgical and other medical procedures worldwide, especially for infants or children [1]. However, increasing evidence has shown that general anesthetics can cause neurotoxicity and hinder brain development in both animals and humans [2,3]. Propofol (2,6-diisopropylphenol) is an intravenous anesthetic widely used in surgery or intensive care units to induce and maintain anesthesia as well as sedation [2]. Despite its advantages in quick onset and recovery after withdrawal, propofol has been verified to induce neurotoxicity, leading to neuronal apoptosis in the brain [4]. Propofol can result in cognitive disorders including impairments in reasoning, planning, learning and memorizing [5]. Moreover, studies have demonstrated that propofol can lead to oxidative stress in the brain, consequently aggravating brain injury [6]. Therefore, a better understanding of the mechanism underlying propofol-induced neurotoxicity is favorable for eliminating the side effects of propofol.

Esketamine is the *S*-enantiomer of ketamine, which is a noncompetitive *N*-methyl-d-aspartate glutamate receptor antagonist with an antidepressant effect [7]. Compared with ketamine, esketamine possesses higher anesthetic potency [8]. Importantly, esketamine has been used to combine with the anesthetic induction of propofol, which mitigates inflammatory response and facilitates recovery of postoperative cognitive functions in elderly surgical patients [9]. A low dose of esketamine promotes the sedation of propofol during endoscopic retrograde cholangiopancreatography [10]. Moreover, esketamine has also been reported to protect against carbon tetrachloride-induced liver damage, oxidative stress and cell apoptosis by activating the Nrf2/HO-1 signaling pathway [11]. It has been recently shown that esketamine attenuates postoperative depression-like symptoms by inhibiting inflammatory responses in the mouse model [12]. However, whether esketamine has an effect on propofol-induced brain injury has not been investigated.

Neurotrophic factors, including brain-derived neurotrophic factor (BDNF), are indispensable regulators for the survival of glial cells and neurons in the central nervous system (CNS) [13]. Initially, BDNF is synthesized as a precursor protein (proBDNF), which then can be processed into a mature BDNF (mBDNF) protein or released as proBDNF that is not modified [14]. Furthermore, by activating different receptor systems, proBDNF and mBDNF induce opposite biological effects [15]. The mBDNF contributes to neuronal survival, differentiation and synaptic plasticity by binding to the tropomyosin receptor kinase B (TrkB) receptor, while proBDNF induces neuronal death by binding to the p75 neurotrophin receptor [16]. The mBDNF/TrkB signaling pathway exerts prominent effects on the development of CNS [17]. Furthermore, accumulating evidence has illustrated the involvement of the mBDNF/TrkB/PI3K (phosphatidylinositide 3-kinase) signaling pathway in the development of brain injury. For example, genistein alleviates the neurotoxicity induced by isoflurane and mitigates spatial learning and memory impairments largely by modulating the BDNF/TrkB/PI3K signaling pathway [18]. Nevertheless, it is unclear whether esketamine is implicated in the activation of the BDNF/TrkB/PI3K signaling.

Herein, we investigated the role of esketamine in propofol-induced brain injury and its potential mechanism. It was hypothesized that esketamine might affect propofol-induced brain injury by regulating certain signaling pathways. The findings might provide a new perspective in protecting against propofol-induced neurotoxicity.

## 2 Materials and methods

### 2.1 Animals and study design

Forty-eight Sprague Dawley rats (male, 6–8 weeks, 240–280 g) were purchased from Vital River (Beijing, China).

The rats were randomly divided into six groups: control, control + esketamine, propofol, propofol + esketamine, propofol + esketamine + IgG and propofol + esketamine + anti-BDNF (*n* = 8/group). Rats in the propofol-treated groups were intraperitoneally injected with 50 mg/kg propofol (Sigma–Aldrich, Louis, MO, USA), followed by an injection of 50 mg/kg propofol again after 1 h when the rat righting reflex recovered [19]. Rats in the control group were administrated with 100 mg/kg intralipid [19]. Thirty minutes after propofol injection, rats in the esketamine-treated groups were intraperitoneally injected with 15 mg/kg esketamine (Cristalia, Brazil) [20]. Normal saline (NS) was used as a control for esketamine.

In some experiments, to examine the role of the BDNF/TrkB/PI3K signaling pathway, anti-BDNF was used for BDNF knockdown assays. Rats were intranasally administrated with anti-BDNF or control IgG (100 μg/kg) [21] using a sterile 26-G Hamilton microsyringe (Hamilton Company, Reno, NV, USA) 30 min before propofol treatment.


**Ethical approval:** The research related to animals use has complied with all the relevant national regulations and institutional policies for the care and use of animals. All animal procedures were conducted following the guidelines for the Care and Use of Laboratory Animals and approved by the Ethics Committee of People’s Hospital of Xinjiang Uygur Autonomous Region. Xinjiang Clinical Research Center for Anesthesia Management (Urumqi, Xinjiang).

### 2.2 Morris water maze (MWM)

To assess the spatial learning and memory of rats, the MWM test was performed according to the previous description [22]. To ensure the objectivity of the results, the experiment was performed by two researchers who had no knowledge of the treatments of the animals. Briefly, the test was implemented in a circular pool (diameter = 90 cm) full of water at 21°C ± 2°C with a hidden platform (diameter = 4.5 cm) located 1 cm below the opaque water in the middle of the third quadrant. The movements of rats were tracked and analyzed with a SLY-WMS MWM analysis system (Sunny Instruments, Beijing, China). The learning ability of rats was tested for 4 days, four times a day with an interval of 30 min. Rats were allowed to swim freely to the platform within 60 s, followed by staying on the platform for 15 s. If the rat failed to find the platform within 60 s, it would be guided to the platform followed by 15 s rest, and the escape latency (time needed for finding the platform) was recorded as 60 s. Average latency was computed post-test. The probe test was carried out to test the spatial memory ability of rats on the fifth day. The platform was removed; rats were placed into the pool and allowed to swim freely for 60 s. The times that rats crossed the original position of the platform and the time that rats stayed in the third quadrant were recorded.

### 2.3 Measurement of oxidative stress markers

After the MWM test, all rats were anesthetized by intraperitoneal injection with 10% 3.5 mL/kg chloral hydrate and then sacrificed by cervical dislocation. Then, rat hippocampal tissues were collected for subsequent analysis. The levels of superoxide dismutase (SOD), lactate dehydrogenase (LDH) and malondialdehyde (MDA) were measured using commercially available assay kits (Nanjing Jiancheng Bioengineering Institute, Nanjing, China) following the manufacturer’s protocols. Reactive oxygen species (ROS) levels in rat hippocampal tissues were analyzed using the 2′,7′-Dichlorofluorescin diacetate (DCFH-DA) ROS Assay Kit (Beyotime, Shanghai, China). When ROS is present in cells, DCFH is oxidized to the fluorescent substance DCF). The fluorescence intensity of DCF is proportional to the ROS level. The cell suspension was obtained by enzymatic digestion of tissue samples and then incubated with DCFH-DA (10 mM) for 30 min at 37°C in the dark. After washing three times to remove DCFH-DA that did not enter the cells, a flow cytometer (BD Biosciences, San Jose, California (CA), USA) was used to measure the fluorescence intensity of DCF. The excitation wavelength was 488 nm, and the emission wavelength was 525 nm.

### 2.4 Hematoxylin-eosin (HE) staining

Rat brains were fixed with 4% paraformaldehyde for 48 h at 4°C, embedded in paraffin wax and cut into sections (5 µm). Tissue sections were baked dry, dewaxed with xylene and then soaked in ethanol in gradient concentrations. Then, the sections were stained with hematoxylin for 5 min and treated with 1% hydrochloric acid for color separation of chromatin and nuclei, followed by staining with 0.5% eosin for 2 min. Next, the sections were dehydrated with ethanol in gradient concentrations, washed with xylene and sealed with resin. The morphological changes of neurons in the hippocampal CA1, CA3 and dentate gyrus (DG) regions were observed under a microscope (Olympus, Tokyo, Japan). A relatively constant number of neurons from each visual field was photographed. The number of cells in the same area in each photo was counted, and the average of three sections was taken as the number of surviving neurons in the tissue sample.

### 2.5 Western blotting

Proteins were extracted from rat hippocampal tissues using radio immunoprecipitation assay buffer (Beyotime) and quantified with a bicinchoninic acid assay kit (Thermo Scientific, Waltham, MA, USA). Equal amounts (20 µg) of tissue samples were separated by 10% sodium dodecyl sulfate polyacrylamide gel electrophoresis and blotted onto the polyvinylidene fluoride membranes (Bio‐Rad, Hercules, CA, USA). After being blocked with 5% defatted milk, the membranes were incubated with primary antibodies against: Bax (ab32503, 1:1,000, Abcam), cleaved (C)-caspase3 (#9661, 1:1,000, Cell Signaling, Danvers, MA, USA), phosphorylated (p)-TrkB (ab229908, 1:1,000, Abcam), TrkB (ab187041, 1:5,000, Abcam), p-PI3K (ab182651, 1:1,000, Abcam), PI3K (ab191606, 1:1,000, Abcam), proBDNF (p1374, 1:400, Sigma–Aldrich), mBDNF (ab108319, 1:1,000, Abcam) and glyceraldehyde-3-phosphate dehydrogenase (ab181602, 1:10,000, Abcam) at 4°C overnight, followed by incubation with the horseradish peroxidase-conjugated goat anti-rabbit secondary antibody (ab7090, 1:1,000, Abcam) at room temperature for 2 h. Eventually, protein bands were visualized with an enhanced chemiluminescence kit (Bio-Rad), and the intensity values were analyzed with C‐Digit software (LI-COR Biosciences, Lincoln, NE, USA).

### 2.6 Statistical analysis

Data are presented as the mean ± standard deviation. Each experiment was performed at least three times. Statistical analysis was performed using SPSS 22.0 software (IBM, Armonk, NY, USA). Differences between the two groups were evaluated by Student’s *t*-test, while those among more than two groups were assessed by one-way analysis of variance (ANOVA) followed by Turkey *post hoc* analysis. For escape latency in the MWM test, two-way ANOVA was used. *p* < 0.05 was considered statistically significant.

## 3 Results

### 3.1 Esketamine mitigates propofol-induced cognitive dysfunction

**Figure 1:**
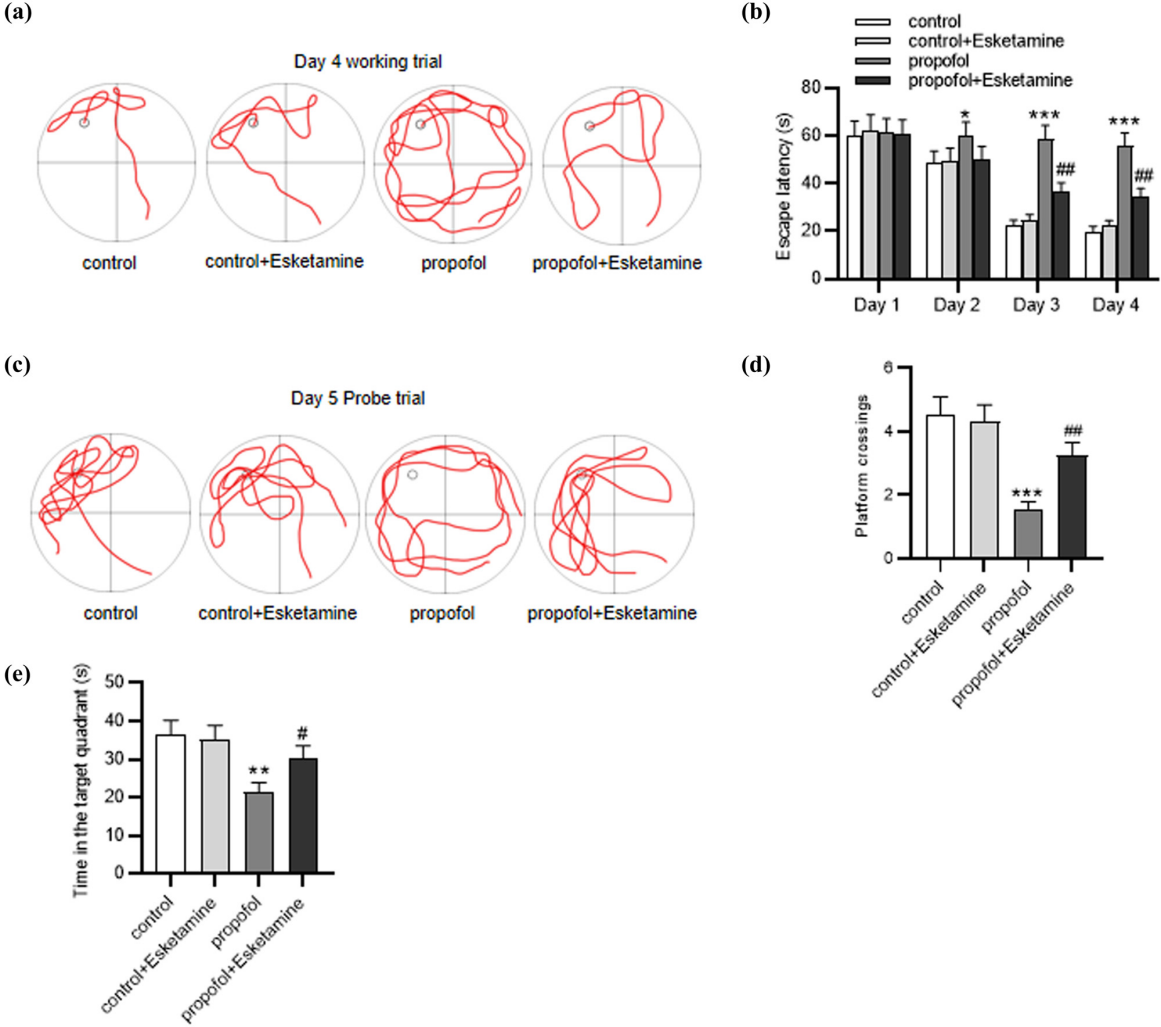
Esketamine mitigates propofol-induced cognitive dysfunction. (a) Representative swimming tracks of rats in the navigation trial of the MWM test. (b) Escape latency of each group with a hidden platform. (c) Representative swimming tracks of rats in the probe trial. (d) The times that the rats crossed the original position of the platform within 60 s. (e) The time that the rats spent in the target quadrant of each group. ^*^ *p* < 0.05, ^**^ *p* < 0.01, ^***^ *p* < 0.001 vs the control group; ^#^ *p* < 0.05, ^##^ *p* < 0.01 vs the propofol group.

First, we tested whether esketamine had an effect on the cognitive functions of rats. The MWM test was used to assess spatial learning and memory. As shown in Figure 1a, rats in propofol-treated groups sought for the hidden platform in a random way compared with those in the control groups, and esketamine treatment was shown to improve this condition. Moreover, on the second day of training, rats in the propofol group exhibited significantly (*p* < 0.05) longer escape latency than those in the control group, and this phenomenon was much more significant (*p* < 0.001) in the following 2 days (Figure 1b), indicating that propofol might induce the impairment in spatial learning ability. In addition, compared with that in the propofol group, the escape latency in the propofol + esketamine group was markedly reduced (*p* < 0.01) (Figure 1b). In the probe trial, rats in the propofol group displayed a random swimming trajectory while those in the propofol + esketamine group displayed an improved manner (Figure 1c). Furthermore, in comparison to the control rats, propofol-administrated rats passed through the original position of the platform fewer times (*p* < 0.001) and spent less time (*p* < 0.01) in the target quadrant (Figure 1d and e), suggesting poor memory retention of the original position of the platform. Notably, simultaneous administration with propofol and esketamine in rats improved (*p* < 0.05) the impaired memory of rats (Figure 1d and e). Collectively, esketamine could mitigate propofol-induced cognitive impairment in rats.

### 3.2 Esketamine relieves propofol-induced oxidative stress

**Figure 2:**
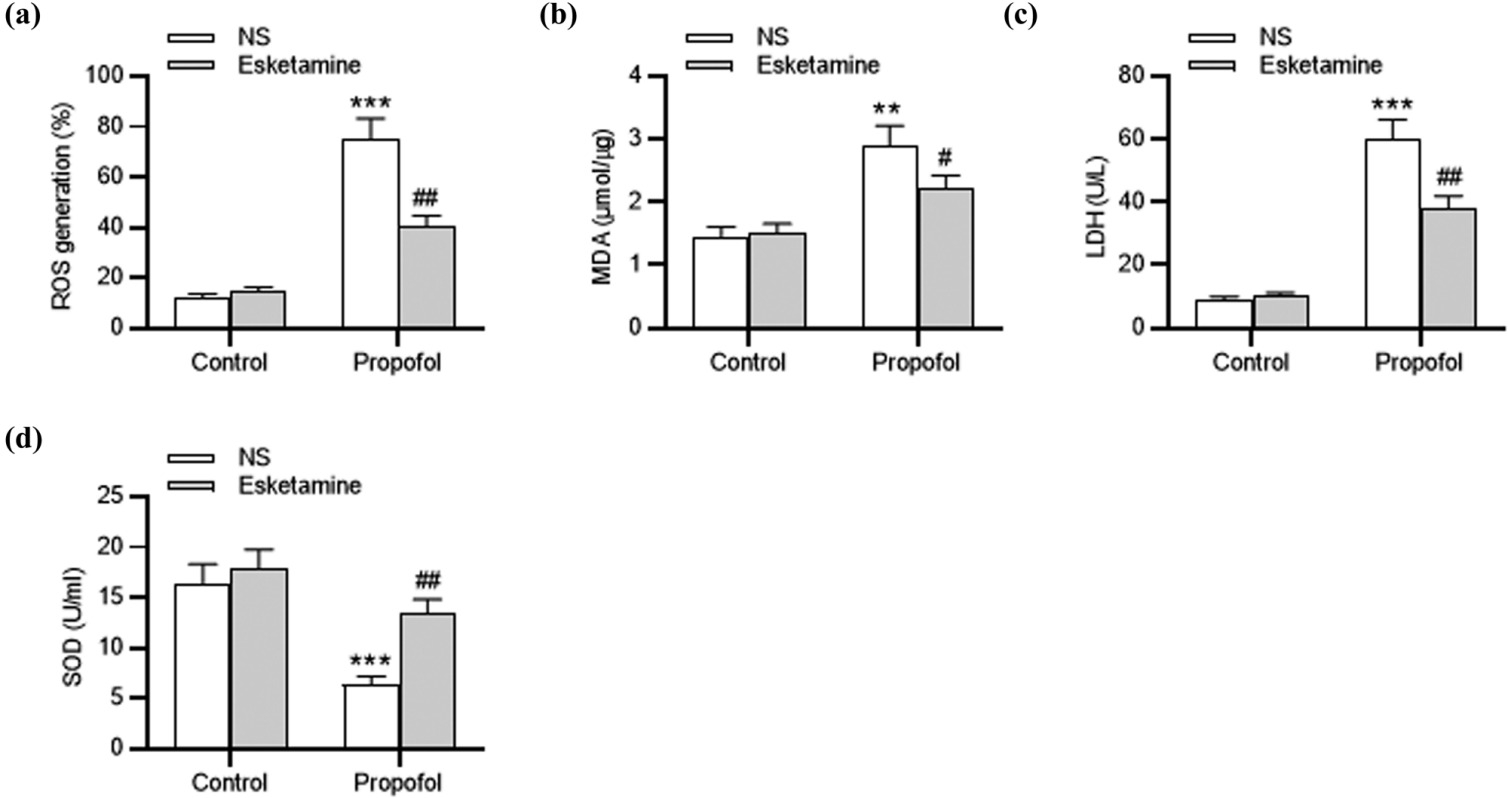
Esketamine attenuates propofol-induced oxidative stress. (a) ROS level in each group measured by relative fluorescence intensity (%) of DCF. (b–d) MDA, LDH and SOD levels in the rat hippocampal tissues evaluated by commercially available assay kits. ^**^ *p* < 0.01, ^***^ *p* < 0.001 vs the control group (treated with NS); ^#^ *p* < 0.05, ^##^ *p* < 0.01 vs the propofol group (treated with NS).

Subsequently, the role of esketamine in regulating oxidative stress was examined. Results revealed that the levels of ROS (*p* < 0.001), MDA (*p* < 0.01) and LDH (*p* < 0.001) were significantly increased in the propofol group, while the level of SOD was reduced (*p* < 0.001), indicating that propofol induced oxidative stress in the brains of rats. Notably, esketamine treatment attenuated (*p* < 0.01) the effects caused by propofol (Figure 2a–d). These suggested that esketamine ameliorated propofol-mediated oxidative stress in rats.

### 3.3 Esketamine alleviates propofol-induced neuronal injury

**Figure 3:**
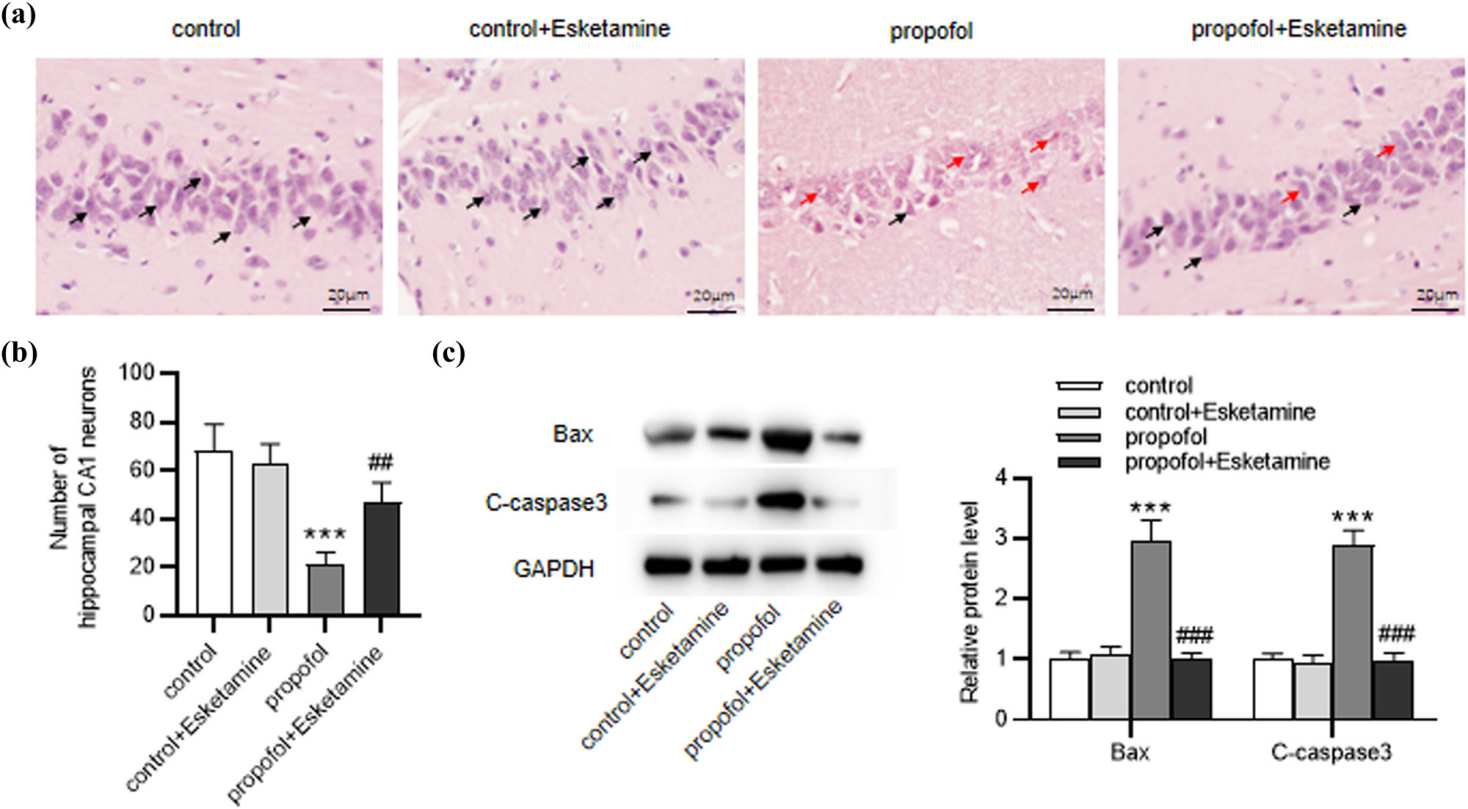
Esketamine alleviates propofol-induced neuronal injury. (a) Representative images of HE staining for detecting the morphological changes of neurons in rat hippocampal CA1 region. The black arrow indicates normal neurons and the red arrow indicates abnormal neurons. (b) The number of neurons in the hippocampal CA1 area. (c) Western blotting for assessing levels of apoptosis-related proteins in each group. ^***^ *p* < 0.001 vs the control group; ^##^ *p* < 0.01, ^###^ *p* < 0.001 vs the propofol group.

HE staining was used for histopathological observation of the rat hippocampi in CA1, CA3 and DG regions. Results show that compared to those in the control group, the pyramidal cells in the propofol group were disorderly arranged, with shrinking cell bodies and nuclei in rat hippocampal CA1 and CA3 areas. Intriguingly, the treatment of esketamine attenuated propofol-induced neuronal injury (Figure 3a and b, Figure S1a and c). Similar results were also observed in the hippocampal DG region (Figure S1b and d). Then, western blotting was used to assess neuronal apoptosis in rat hippocampi. As displayed by the results, the levels of apoptosis-related proteins (Bax, C-caspase3) were elevated (*p* < 0.001) in the propofol group but were decreased (*p* < 0.001) in the propofol + esketamine group (Figure 3b), indicating that esketamine alleviated propofol-stimulated neuronal apoptosis. The above data showed the protective role of esketamine in propofol-mediated brain injury.

### 3.4 Esketamine attenuates propofol-mediated inhibition on mBDNF/TrkB/PI3K signaling

**Figure 4:**
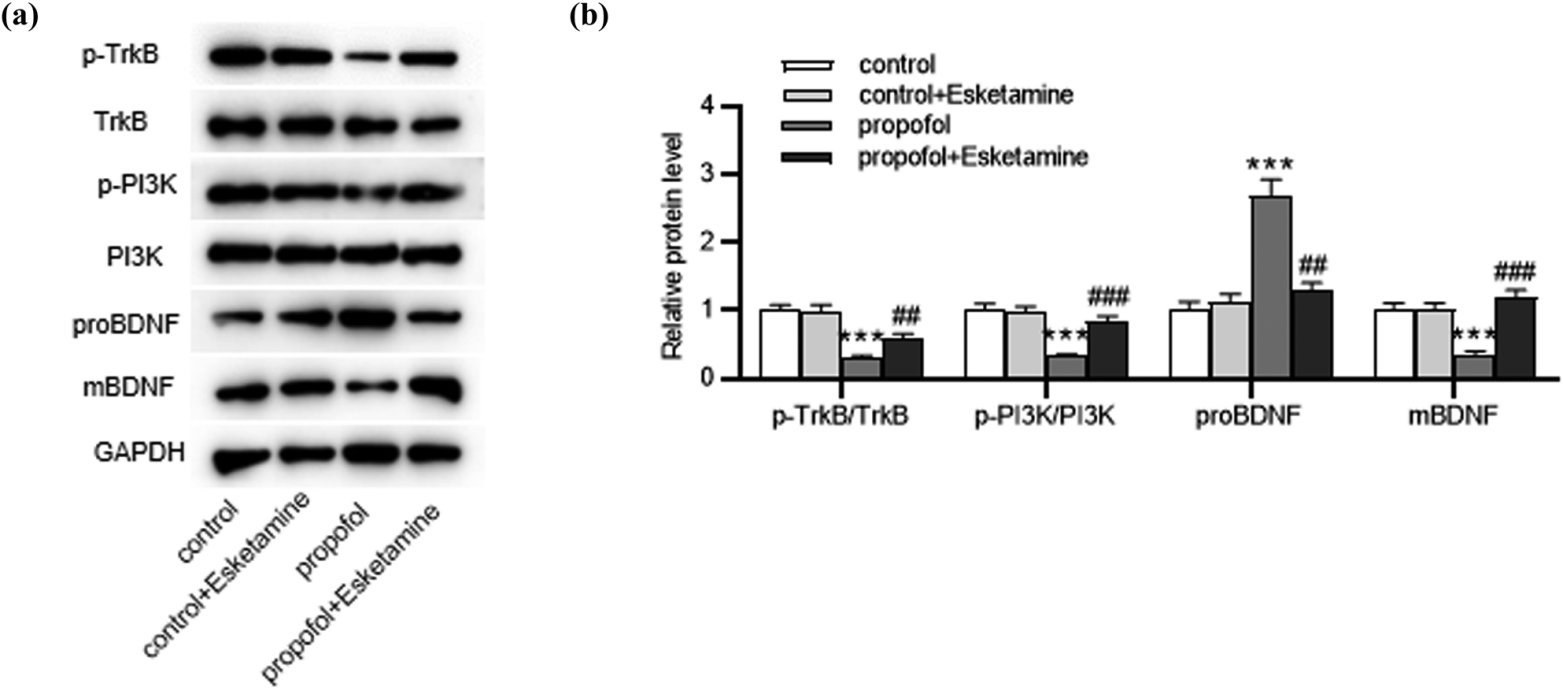
Esketamine attenuates propofol-mediated inhibition of mBDNF/TrkB/PI3K signaling. (a and b) Western blotting for evaluating the levels of mBDNF/TrkB/PI3K signaling-associated proteins in each group. ^***^ *p* < 0.001 vs the control group; ^##^ *p* < 0.01, ^###^ *p* < 0.001 vs the propofol group.

To understand the potential mechanism of esketamine underlying propofol-induced brain injury, we detected the effect of esketamine on the mBDNF/TrkB/PI3K signaling pathway. As revealed by western blotting, the ratios of p-TrkB to total TrkB and p-PI3K to total PI3K were decreased (*p* < 0.001) in the propofol-treated group but were enhanced (*p* < 0.001) in propofol and esketamine-treated groups (Figure 4a and b). Notably, the levels of proBDNF and mBDNF proteins displayed opposite trends in rats treated with propofol; mBDNF protein, which promotes neuronal survival, was downregulated (*p* < 0.001) in propofol-treated rats but was elevated (*p* < 0.001) after the administration of esketamine (Figure 4a and b). These suggested that esketamine activated the mBDNF/TrkB/PI3K signaling in propofol-administrated rats.

### 3.5 Inhibition of mBDNF/TrkB/PI3K signaling attenuates the suppressive effect of esketamine on propofol-induced neuronal apoptosis

**Figure 5:**
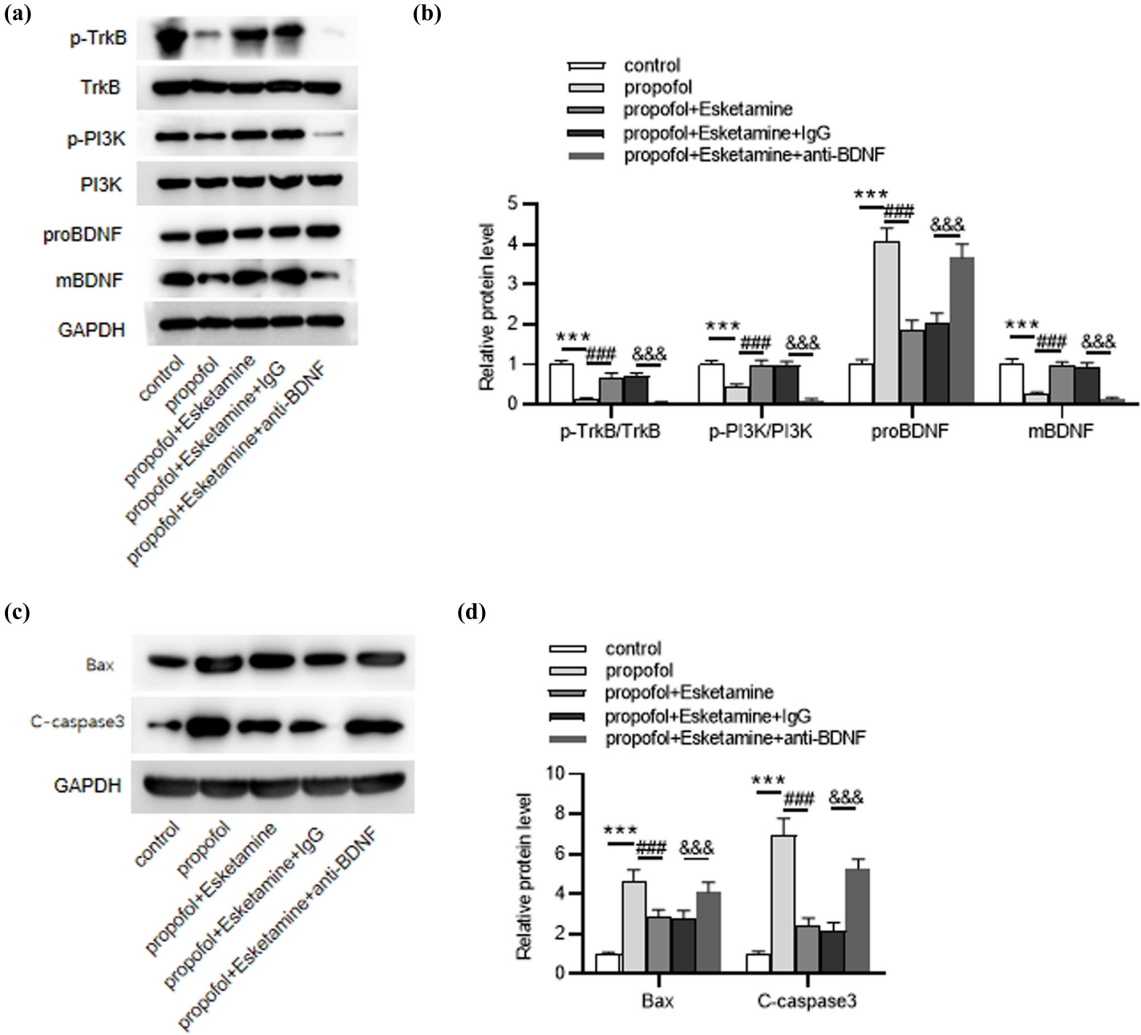
Anti-BDNF reverses esketamine-mediated neuroapoptosis improvement in propofol-treated rats. (a and b) Western blotting for evaluating the levels of mBDNF/TrkB/PI3K signaling-associated proteins in each group. (c and d) Western blotting for assessing the levels of pro-apoptosis proteins in each group. ^***^ *p* < 0.001 vs the control group. ^###^ *p* < 0.001 vs the propofol group. ^&&&^ *p* < 0.001 vs the propofol + Esketamine + IgG group.

Then, we investigated the role of mBDNF/TrkB/PI3K signaling in propofol-induced neuronal injury. Notably, esketamine-triggered phosphorylation of p-TrkB and p-PI3K as well as upregulation of mBDNF were abated (*p* < 0.001) by anti-BDNF treatment in propofol-administrated rats (Figure 5a and b), indicating that BDNF knockdown inhibited the mBDNF/TrkB/PI3K signaling pathway in propofol-treated rats. Moreover, western blotting revealed that compared to the control IgG, anti-BDNF treatment significantly (*p* < 0.001) increased the levels of pro-apoptotic proteins (Bax and C-caspase3) in propofol- and esketamine-administrated rats (Figure 5c and d). The above data indicated that esketamine improved propofol-induced neuroapoptosis by activating the mBDNF/TrkB/PI3K signaling pathway.

## 4 Discussion

Emerging evidence has demonstrated that general anesthetics can induce neurotoxicity, consequently leading to cognitive disorders and neuroapoptosis in the brains of animals and humans [23]. As one of the most commonly used anesthetics, propofol has a great anesthetic effect in the clinic; however, increasing evidence has verified that propofol can induce acute neurotoxicity and cause long-term neurological damage [24]. Based on this, we established a rat model of brain injury induced by propofol in this study, aiming to have a better understanding of the mechanism underlying propofol-induced neurotoxicity.

Esketamine is the S-enantiomer of ketamine with an antidepressant effect, and in terms of anesthesia, esketamine is more potent than ketamine [25]. A combination of esketamine and propofol can promote the recovery of postoperative cognitive functions in elderly surgical patients [9]. Here, we investigated the role of esketamine involved in propofol-induced brain injury in rats. Behavioral tests revealed that propofol treatment could impair the spatial learning and memory of rats, but the treatment of esketamine was shown to attenuate propofol-induced cognitive impairments. Furthermore, we examined the role of esketamine in oxidative stress. Previous studies have demonstrated that propofol can lead to oxidative stress and cell apoptosis [26,27]. Oxidative stress is caused by the imbalance between ROS production and elimination [28]. In addition, an imbalance between oxidants such as LDH and MDA and antioxidants such as SOD is also responsible for oxidative stress [29]. Oxidative stress in brains leads to neuroinflammation, consequently aggregating brain injury [30]. In this study, propofol-treated rats exhibited increased levels of ROS, MDA and LDH and a decreased level of SOD, confirming that propofol could induce oxidative stress in rat brain tissues. Intriguingly, the effects induced by propofol were partially reversed by esketamine, indicating that esketamine could relieve propofol-mediated oxidative stress. Consistent with previous studies, our results revealed that propofol caused brain injury and induced neuronal apoptosis in rats. However, simultaneous administration of propofol and esketamine significantly alleviated the neuronal injury caused by propofol. Collectively, esketamine can attenuate propofol-induced cognitive dysfunction, oxidative stress and neuronal apoptosis in rats.

The mBDNF/TrkB/PI3K pathway is considered the main signaling pathway implicated in cerebral ischemia-mediated neuronal apoptosis [31]. Previous studies have verified that BDNF exerts anti-inflammatory and neuroprotective effects and contributes to neuronal development, survival and plasticity in the CNS by binding to its receptor TrkB [32]. Besides, PI3K signaling is essential for cell proliferation and survival [33]. It was reported that edaravone attenuated propofol-induced neurotoxicity by regulating mBDNF/TrkB/PI3K signaling [34]. Here, we detected whether esketamine affected propofol-mediated brain injury via the mBDNF/TrkB/PI3K signaling pathway. The results showed that propofol inhibited the activation of the signaling pathway, but esketamine reversed this effect in rat hippocampal tissues. Moreover, the knockdown of BDNF partially reversed esketamine-mediated improvement in neuroapoptosis in propofol-induced rats, indicating that esketamine suppressed propofol-induced neuronal apoptosis by activating the mBDNF/TrkB/PI3K signaling pathway.

In conclusion, we explored the role and potential mechanism of esketamine underlying propofol-induced brain injury in rats. The results reveal that esketamine attenuates propofol-mediated cognitive dysfunction, oxidative stress and neuronal injury in rats. Moreover, esketamine improves propofol-induced neuroapoptosis by activating the mBDNF/TrkB/PI3K signaling pathway in rat hippocampal tissues. The findings might provide a new perspective for eliminating the neurotoxicity induced by propofol anesthesia. In addition, more investigations are needed in the future to further elucidate the functions and mechanisms of esketamine in propofol-triggered brain injury.

## Supplementary Material

Supplementary Figure
