# Supplementary material for: Esketamine improves propofol-induced brain injury and cognitive impairment in rats
Source: Transl Neurosci. 2022 Dec 6;13(1):430–9. doi: 10.1515/tnsci-2022-0251 (PMC9730546; doi:10.1515/tnsci-2022-0251)
Supplement: Supplementary Figure [file tnsci-2022-0251-sm.pdf]

Supplementary materials

1 Supplementary information

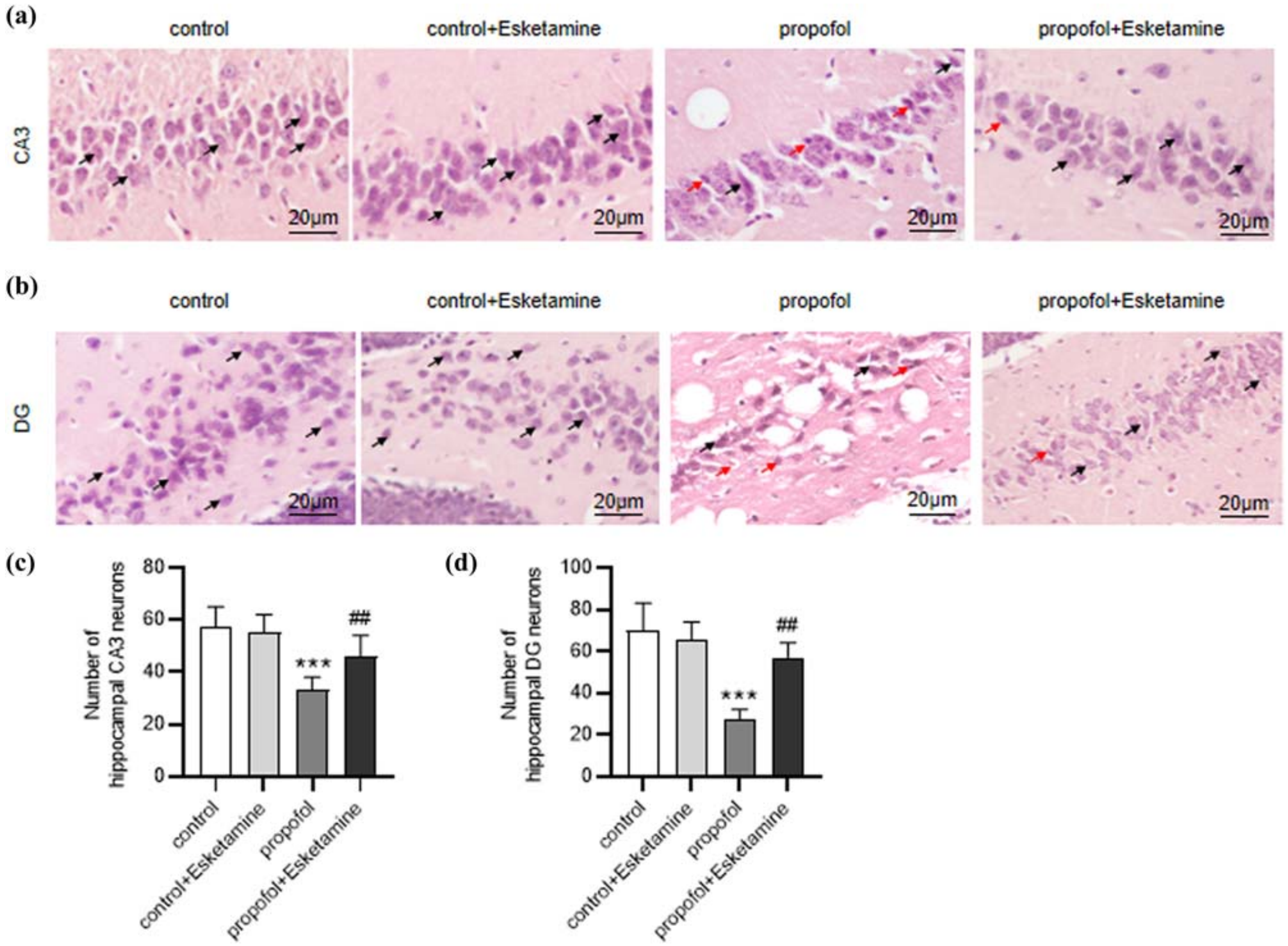

Figure S1: A-B. Representative images of HE staining for analyzing morphological changes of neurons in hippocampal CA3 region (a) and DG region (b). Black arrow: normal neurons, red arrow: abnormal neurons. C-D. The number of neurons in hippocampal CA3 region (c) and DG region (d). ***p˂0.001 vs. the control group. ##p˂0.01 vs. the propofol group.
